# Intralesional Endoscopy and Septectomy as a Diagnostic Tool and Treatment Method for Lymphatic Malformations

**DOI:** 10.1055/s-0037-1606388

**Published:** 2017-10-15

**Authors:** Anne-Sophie Holler, Jan Gödeke, Veronika Engel, Oliver J. Muensterer

**Affiliations:** 1 Department of Pediatric Surgery, University Medicine of the Johannes Gutenberg University Mainz, Mainz, Germany

## Case Report

Sclerotherapy and surgery are both effective treatment methods for lymphatic malformations. 1 2 However, recurrence due to incomplete resection is a common problem, often necessitating multiple treatment sessions. 2 Intralesional endoscopy has been described as a diagnostic approach and potential therapeutic tool. 1 3

**Fig. 1:**
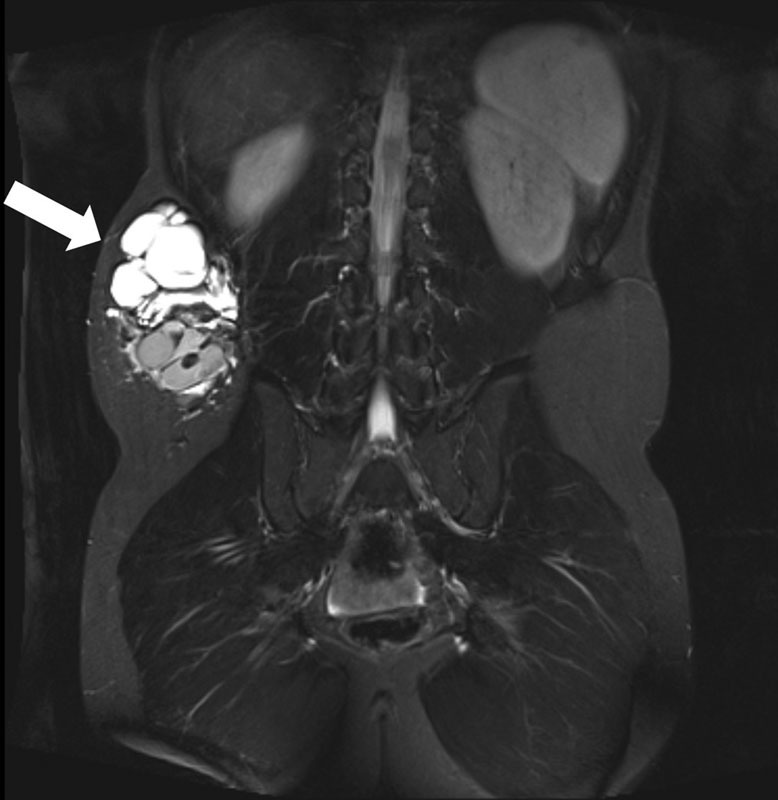
Coronal T2 magnetic resonance imaging (MRI) with a subcutaneous mixed macro-/microcystic lymphatic malformation on the patient's right flank (white arrow).

**Fig. 2:**
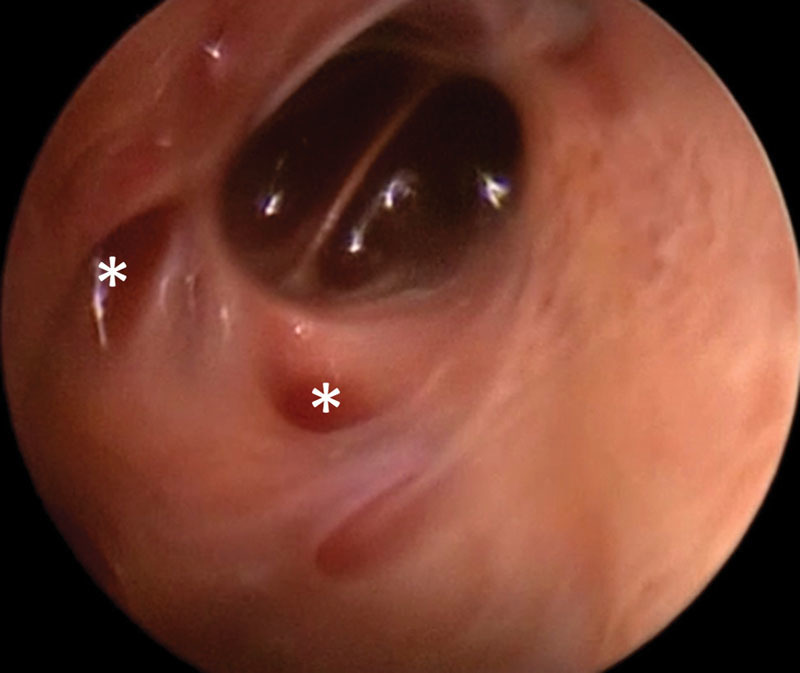
Intraoperative image showing a macrocystic cavity and many small fluid-filled cysts (asterisks), which are separated by thin septae.

We report a case of a 12-year-old male patient who presented with a mainly subcutaneous mixed lymphatic malformation located on the right flank ( Fig. 1 ). After suffering a direct trauma, the lesion had increased markedly in size and surgical intervention was indicated due to the associated pain. Intralesional endoscopy was performed that showed a mixed macro-/microcystic lymphatic malformation with hemorrhage ( Fig. 2 ). Intercystic septa were dissected under endoscopic visualization. At the end of the procedure, a single macrocystic cavity had been artificially created. Picibanil (OK-432) was inserted into the cavity and left in situ for 24 hours ( Video 1 ). The patient had no visible swelling, no pain, and merely two small, well-healed scars at 2 months of follow-up.

**Video 1** Under sonographic guidance, the largest cyst of the lymphatic malformation was cannulated, and an 8 French pigtail catheter was advanced into the cyst over a guidewire. The cyst was filled with 0.9% sodium chloride solution to facilitate the placement of two 3-mm trocars into the cyst. Intralesional endoscopy showed a mixed macro-/microcystic lymphatic malformation with hemorrhage. Dissection of intercystic septa was performed under endoscopic visualization using hook electrocautery and blunt dissection. At the end of the procedure, a single macrocystic cavity had been artificially created. Picibanil (OK-432) was instilled through the formerly inserted pigtail catheter into the now solitary cyst and left in situ for 24 hours.

Intralesional endoscopy and intercystic septectomy constitute an interesting novel approach for the diagnosis and treatment of mixed lymphatic malformations. By creating a single, communicating cavity, the efficacy of subsequent sclerotherapy may be increased, and thus the need for multiple treatment sessions may be avoidable.

## New Insights and the Importance for the Pediatric Surgeon

Current treatment options for lymphatic malformations are expectant management, sclerotherapy, and surgical resection. Intralesional endoscopy and intercystic septectomy constitute a promising supplementary treatment method for subcutaneous lymphatic malformations. By creating a single, communicating cavity, the efficacy of subsequent sclerotherapy may be increased, and thus the need for multiple treatment sessions may be avoidable.
